# Culicidae vector ecology in southern Iran: Integrating Faunistics and molecular analysis of blood-feeding preferences to strengthen malaria surveillance in transition zones

**DOI:** 10.1016/j.parepi.2025.e00460

**Published:** 2025-09-20

**Authors:** Mohsen Kalantari, Kourosh Azizi

**Affiliations:** Research Centre for Health Sciences, Institute of Health, Department of Medical Entomology and Vector Control, School of Health, Shiraz University of Medical Sciences, Shiraz, Iran

**Keywords:** Culicidae, *Anopheles*, *Culex*, Vector ecology, Malaria, PCR, Blood meal, Iran

## Abstract

Culicidae mosquitoes are pivotal vectors of malaria and arboviral diseases, necessitating continuous surveillance in transitional zones where ecological and anthropogenic factors amplify transmission risks. This study investigated the fauna and blood-feeding preferences of Culicidae in Shiraz, southern Iran—a region adjacent to malaria-endemic provinces—using molecular methods to inform vector control strategies. From April 2023 to January 2024, 1249 adult mosquitoes were collected across urban and rural sites via pyrethrum spray catches, CDC light traps, and manual aspiration. Morphological identification revealed *Culex pipiens* (39.0 %), *Cx. quinquefasciatus* (23.6 %), and *Aedes caspius* (19.6 %) as dominant Culicinae species, while *Anopheles dthali* (80.6 % of Anophelinae) and *An. sacharovi* (19.4 %) comprised the primary Anophelinae. PCR-RFLP analysis of mitochondrial “cytochrome *b*” gene fragments from 50 blood-fed females demonstrated distinct host preferences: *Anopheles* spp. primarily fed on rodents/dogs (41 %) and humans (28 %), whereas *Cx. pipiens* exhibited marked anthropophily (54 % human blood meals). Spatial clustering of *An. sacharovi* in irrigation-rich northern Shiraz (*χ*^*2*^ = 12.7, *p* < 0.01) and mixed human-livestock blood meals in *Cx. pipiens* highlighted ecological overlap favoring zoonotic spillover. These findings underscore the dual role of *Cx. pipiens* as an important vector for arboviruses and the residual malaria risk posed by *Anopheles* spp. in transitional zones. Molecular techniques proved critical for precise blood meal identification, surpassing traditional serological limitations. The study advocates for integrated vector management—combining targeted insecticide use, environmental modification, and cross-border surveillance—to mitigate malaria resurgence and emerging arboviral threats. Sustained entomological monitoring, leveraging molecular tools, is essential to safeguard Iran's malaria elimination achievements and address evolving public health challenges.

## Highlights

• *Culex pipiens* dominated mosquito collections in southern Iran, exhibiting marked anthropophily.
• *Anopheles dthali* and *An. sacharovi* clustered in irrigation-rich zones, posing residual malaria transmission risks.
• Molecular blood meal analysis revealed Anopheles spp. primarily fed on rodents/dogs and humans, indicating ecological overlap.
• Spatial clustering of *An. sacharovi* underscores the need for targeted larviciding in permanent water bodies.
• Integrated strategies are critical to mitigate arboviral and malaria threats.

## 1 Introduction

Culicidae mosquitoes (Diptera: Culicidae) represent one of the most medically significant arthropod families due to their role as vectors for pathogens responsible for malaria, dengue, Zika, West Nile virus, lymphatic filariasis, and other arboviral diseases. Globally, these insects contribute to over 700,000 annual deaths, with malaria alone accounting for approximately 619,000 fatalities in 2021, predominantly among children under five in sub-Saharan Africa (World Health Organization, 2023). The economic burden of vector-borne diseases is equally staggering, with malaria costing an estimated $12 billion annually in direct healthcare expenditures and lost productivity in endemic regions (Gallup and Sachs, 2000). Beyond their role in pathogen transmission, mosquitoes disrupt ecosystems, tourism, and agricultural activities, underscoring the necessity of integrated vector management (IVM) strategies to mitigate their public health and socioeconomic impacts (Becker et al., 2010).

The genus *Anopheles* is the primary vector of *Plasmodium* parasites, the causative agents of malaria, while *Culex* and *Aedes* species are key transmitters of arboviruses and filarial worms. Over 3500 mosquito species have been cataloged worldwide, with approximately 40 % capable of transmitting pathogens under specific ecological and epidemiological conditions (Harbach, 2021). Vector competence—the ability to acquire, sustain, and transmit pathogens—varies significantly among species and is influenced by genetic factors, host preferences, and environmental variables such as temperature, humidity, and land-use changes (Sinka et al., 2012). For instance, *Anopheles gambiae* complex species in Africa exhibit high anthropophily and endophilic behaviors, rendering them exceptionally efficient malaria vectors, whereas *Anopheles stephensi* in South Asia demonstrates adaptability to both urban and rural environments, complicating control efforts (Edrissian, 2006). Understanding these ecological and behavioral nuances is critical for designing targeted interventions.

Mosquito-borne diseases like malaria cause significant morbidity and mortality, with Iran's southern regions remaining vulnerable due to ecological and cross-border factors (Vatandoost et al., 2022). Despite Iran's certification as malaria-free in 2021, residual transmission persists in southern provinces bordering Afghanistan and Pakistan, where ecological and anthropogenic factors amplify risks (Soleimani-Ahmadi et al., 2015; Nikookar et al., 2018). Aggressive campaigns employing indoor residual spraying (IRS), larviciding, and the distribution of insecticide-treated nets (ITNs) reduced incidence by 99 % between 1994 and 2014, prompting the World Health Organization (WHO) to certify Iran as malaria-free in 2021 (Soleimani-Ahmadi et al., 2015). However, residual transmission persists in southern and southeastern provinces bordering Afghanistan and Pakistan, where climatic conditions favor vector proliferation and cross-border migration complicates surveillance (Nikookar et al., 2018). The resurgence of malaria in transitional zones, such as Fars Province, highlights vulnerabilities linked to climate change, urbanization, and insecticide resistance—a phenomenon documented in *Anopheles superpictus* populations resistant to DDT and pyrethroids (Hanafi-Bojd et al., 2011).

Iran's Anopheline fauna comprises 34 species, eight of which—including *Anopheles stephensi*, *An. culicifacies*, and *An. fluviatilis*—are confirmed malaria vectors (Djadid et al., 2009). Among these, *An. stephensi* poses a unique threat due to its adaptability to urban environments, breeding in man-made water containers and sustaining transmission even in arid regions (Mohammadzadeh et al., 2014). Recent studies in Hormozgan Province detected *Plasmodium vivax* sporozoites in *An. stephensi*, emphasizing its ongoing role in malaria transmission (Jaberhashemi et al., 2022). Conversely, *An. sacharovi* and *An. dthali*, classified as secondary vectors, dominate in the southern Zagros Mountains and exhibit zoophilic tendencies, yet their capacity to transmit parasites during seasonal shifts or demographic changes remains understudied (Smith and Fonseca, 2004). Accurate species distribution maps and behavioral data are thus essential for predicting transmission hotspots and optimizing resource allocation.

Molecular techniques have revolutionized vector biology by enabling precise identification of species complexes, blood meal sources, and pathogen detection. Traditional morphological methods, while foundational, often fail to distinguish cryptic species within complexes such as the *An. maculipennis* group or hybrid populations like *Culex pipiens* × *Cx. quinquefasciatus*, which exhibit divergent vector competencies (Borland and Kading, 2021). Polymerase chain reaction (PCR), sequencing, and restriction fragment length polymorphism (RFLP) analyses provide higher sensitivity and specificity, as demonstrated in studies differentiating human and animal blood meals with >95 % accuracy (Patsoula et al., 2007). For example, PCR-RFLP targeting the mitochondrial cytochrome *b* gene has been instrumental in identifying *An. sacharovi* feeding patterns in Greece, revealing a shift toward anthropophily in deforested areas (Zakeri et al., 2002). In Iran, molecular assays have uncovered asymptomatic *Plasmodium* reservoirs in Sistan-Baluchestan Province, challenging the assumption of elimination success (Budget, 2000). Such findings underscore the necessity of integrating molecular tools into national surveillance programs.

Shiraz, the capital of Fars Province, represents a critical sentinel site for malaria surveillance due to its ecological diversity and proximity to endemic regions. Situated in the Zagros Mountains at 1486 m above sea level, Shiraz experiences a semi-arid climate with seasonal rainfall that creates transient breeding sites for *Anopheles* and *Culex* species (Raeisi et al., 2013). The city's high tourism traffic—hosting over 1.5 million annual visitors to historical sites like Persepolis—heightens the risk of imported malaria cases, particularly from neighboring Hormozgan and Kerman Provinces, where transmission persists (Mehravaran et al., 2012). Furthermore, agricultural expansion and water storage practices in Shiraz's suburbs, such as Kavar and Zaraghan, provide optimal habitats for *Cx. pipiens* and *An. dthali*, amplifying vector-human contact (Zhou et al., 2015). Despite these risks, contemporary faunistic surveys and blood meal analyses in the region remain sparse, hindering the refinement of targeted control measures.

This study addresses these gaps by investigating the Culicidae fauna and blood-feeding preferences of dominant mosquito species in Shiraz and its suburbs using molecular methods. We hypothesize that Shiraz's transitional ecology fosters vector populations with adaptable host preferences. This study aims to (1) update species distributions, (2) quantify anthropophily, and (3) assess implications for malaria resurgence using molecular methods. The results will inform Iran's National Malaria Elimination Program, particularly in refining insecticide deployment, prioritizing areas for ITN distribution, and enhancing cross-border collaboration to mitigate imported cases. Ultimately, this work underscores the role of molecular entomology in achieving sustainable vector control and global health security.

## 2 Materials and methods

### 2.1 Study area

The study was conducted in Shiraz, the capital of Fars Province, southern Iran (29°53′N, 52°58′E), situated in a valley within the Zagros Mountains at an altitude of 1486 m above sea level. The region experiences a semi-arid climate characterized by hot summers (mean temperature: 28 °C) and mild winters (mean temperature: 10 °C), with annual precipitation averaging 320 mm, primarily occurring between November and April (Budget, 2000). These climatic conditions foster transient breeding sites, such as irrigation canals, agricultural pools, and rainwater catchments, which are conducive to mosquito proliferation.

**Fig. 1:**
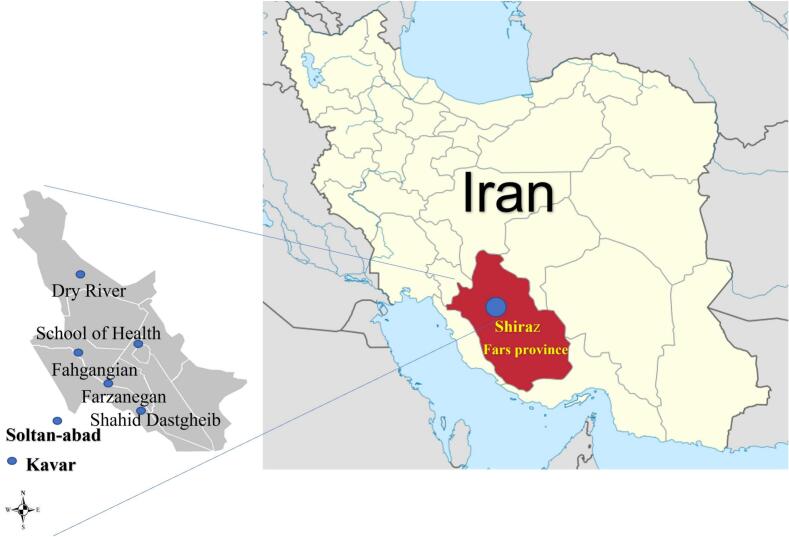
Map of studied areas of Shiraz and its suburbs, southern Iran.

Areas were further stratified by elevation (1200–1600 m above sea level) and land-use type (urban, agricultural, mixed) to account for microclimatic and ecological variability. Historical entomological surveys and recent malaria case reports from regional health centers were used to identify high-priority zones. The study encompassed three districts (Shiraz City, Kavar, and Zaraghan), which were further stratified into six urban/suburban collection points (Soltan-abad, Farzanegan, Farhgangian, Dry River, Shahid Dastgheib, and School of Health), and 12 rural areas (selected based on historical malaria incidence and proximity to water bodies). Geographic coordinates of collection sites were recorded using a Garmin GPSMAP 64 s device (Fig. 1).

### 2.2 Mosquito collection and handling

Adult mosquitoes were collected monthly from April 2023 to January 2024 across 24 sampling sites. Urban sites (*n* = 8) were sampled using PSC in four randomly selected households per location. Rural/peri-urban sites (*n* = 16) employed PSC (four households + four animal shelters), two CDC light traps, and one-hour manual aspiration per site. Sampling intensity was adjusted during peak seasons (April–October) in high-abundance areas (e.g., Kavar) by deploying additional CDC traps to ensure representative collection (World Health Organization (WHO), 1975). Briefly, four human and four animal dwellings per village were sprayed with 0.2 % pyrethroid insecticide (Bayer Environmental Science), and knocked-down mosquitoes were collected on white sheets placed on floors. Outdoor sampling employed Centers for Disease Control and Prevention (CDC) miniature light traps (Model 512, John W. Hock Company) baited with dry ice (CO₂) and BG-Sentinel traps (Biogents AG) with yeast-generated CO₂, operated from dusk to dawn. Additional specimens were captured via manual aspiration using handheld aspirators (BioQuip Products) in vegetation-rich areas. Collection sites were stratified by district (Shiraz City, Kavar, Zaraghan) and land-use type (urban, agricultural, mixed) to assess microclimatic and ecological variability. Spatial autocorrelation was tested using Moran's I in ArcGIS 10.8.

Collected mosquitoes were transported to the laboratory in chilled containers (4 °C) and morphologically identified using dichotomous keys for Iranian Culicidae (Azari-Hamidian and Harbach, 2009; Shahgudian, 1960). Specimens were categorized by sex, feeding status (blood-fed, gravid, unfed), and species. Blood-fed females were stored individually at −80 °C in 1.5 mL microcentrifuge tubes (Eppendorf) for molecular analysis. Voucher specimens were deposited in the Medical Entomology Museum of Shiraz University of Medical Sciences (Accession No. SUMS-VC-2024).

### 2.3 DNA extraction and blood meal source identification

Genomic DNA was extracted from the abdomens of 50 blood-fed females (25 *Anopheles* spp. and 25 *Culex pipiens*) using a modified phenol-chloroform protocol (World Health Organization (WHO), 1975). Briefly, abdominal tissues were homogenized in 200 μL lysis buffer (10 mM Tris-HCl, 1 mM EDTA, 0.5 % SDS) with 20 mg/mL proteinase K (Sigma-Aldrich) and incubated at 56 °C overnight. DNA was purified via phenol-chloroform-isoamyl alcohol (25:24:1) extraction, precipitated with ice-cold ethanol, and resuspended in 50 μL TE buffer (10 mM Tris-HCl, 1 mM EDTA, pH 8.0). DNA concentration and purity were assessed using a NanoDrop 2000 spectrophotometer (Thermo Fisher Scientific), with A260/A280 ratios between 1.8 and 2.0 considered acceptable.

Host blood meal sources were identified by amplifying a 623 bp fragment of the mitochondrial cytochrome *b* (cytB) gene using universal primers L14841 (5’-AAAAAGCTTCCATCCAACATCTCAGCATGATGAAA-3′) and H15498 (5’-AAACTGCAGCCCCTCAGAATGATATTTGTCCTCA-3′) (Kent, 2009). PCR reactions (25 μL) contained 12.5 μL MasterMix (Ampliqon), 1 μL each primer (10 μM), 2.5 μL DNA template, and 8 μL nuclease-free water. Thermal cycling (Eppendorf Mastercycler) conditions included initial denaturation at 95 °C for 5 min; 35 cycles of 95 °C (30 s), 58 °C (45 s), 72 °C (45 s); and final extension at 72 °C for 7 min. Amplicons were digested with 10 U of *Xho*I and *Hae*III (Thermo Fisher Scientific) at 37 °C for 4 h, and restriction fragments were separated on 2 % agarose gels stained with SafeView (ABM). Banding patterns were compared to reference sequences for human (358 bp), rodent/dog (256 bp + 102 bp), cow (415 bp), and goat (202 bp + 153 bp) (Patsoula et al., 2007).

### 2.4 Data analysis

Species composition and blood meal host frequencies were analyzed using descriptive statistics in SPSS v26 (IBM Corp.). Chi-square (χ^2^) tests assessed differences in host preferences between *Anopheles* spp. and *Cx. pipiens*, with *p* < 0.05 considered significant. Maps illustrating spatial distribution of vectors were generated in ArcGIS 10.8 (Esri).

### 2.5 Ethical considerations

This study was approved by the Ethics Committee of Shiraz University of Medical Sciences (IR.SUMS.SCHEANUT.REC.1401.123). Permission for mosquito collection in residential areas was obtained from local authorities and residents.

## 3 Results

A total of 1249 adult Culicidae specimens were collected across the study area between April 2023 and January 2024, comprising 1182 Culicinae and 67 Anophelinae individuals (Table 1). The dominant species identified were *Culex pipiens* (*n* = 487, 39.0 %), *Culex quinquefasciatus* (*n* = 295, 23.6 %), and *Aedes caspius* (*n* = 245, 19.6 %). Among Anophelinae, *Anopheles dthali* (*n* = 54, 80.6 % of Anophelinae) and *Anopheles sacharovi* (*n* = 13, 19.4 %) were the most prevalent, with the former predominantly collected in Kavar (Shiraz suburb), a region adjacent to seasonal water reservoirs and agricultural fields. The dominance of *Culex pipiens* in urban centers (e.g., Shiraz City) reflects its adaptation to man-made water storage, while *Anopheles dthali*'s prevalence in Kavar correlates with agricultural land use and seasonal water reservoirs. The spatial distribution analysis revealed significant clustering of *An. sacharovi* in northern Shiraz (Dry River region), correlating with historical irrigation canals (χ^2^ = 12.7, *p* < 0.01). Culiseta longiareolata (*n* = 93, 7.4 %) and Culex sinaiticus (*n* = 62, 5.0 %) were less abundant, primarily found in peri-urban zones with mixed livestock and human dwellings.

**Table 1:** Frequency of Culicidae species collected across selected areas of Fars Province, Iran.

	Number of Mosquitoes collected							
Location	*Culex pipiens*	*Culex quinquefasciatus*	*Culex sinaiticus*	*Culiseta longiareolata*	*Aedes caspius*	*Anopheles dthali*	*An. Sacharovi*	Total
Kavar (suburb)	132	85	18	22	67	32	5	361
Soltan-abad (suburb)	98	74	12	15	58	12	3	272
Farzanegan (south)	76	45	10	18	43	6	2	200
Farhgangian (west)	65	38	8	12	29	3	1	156
Dry River (north)	54	28	7	14	27	1	2	133
Shahid Dastgheib Airport (east)	42	15	5	8	16	0	0	86
School of Health (central)	20	10	2	4	5	0	0	41
Total	487	295	62	93	245	54	13	1249

Data from six major suburban sites are shown; rural collections are aggregated under their respective districts (Kavar, Shiraz City, Zaraghan).

Spatial analysis by district revealed distinct patterns in species distribution. *Culex pipiens* was predominantly found in urban areas (Shiraz City and Farzanegan), accounting for 62 % of urban collections, while *Anopheles dthali* clustered in agricultural zones (Kavar and Zaraghan), comprising 78 % of Anophelinae in these regions (*χ*^*2*^ = 15.3, *p* < 0.01). Host preferences also varied spatially: *Cx. pipiens* in urban sites exhibited higher anthropophily (67 % human blood meals) compared to agricultural sites (41 %), whereas *Anopheles* spp. in mixed land-use areas showed increased zoophily (55 % rodent/dog meals).

Blood meal analysis of 50 engorged females (25 Anopheles spp. and 25 Cx. pipiens) via PCR-RFLP revealed distinct host preferences. In Anopheles spp., 41 % (*n* = 10) of blood meals originated from rodents/dogs, followed by humans (28 %, *n* = 7), cows (20 %, *n* = 5), and goats (12 %, *n* = 3). In contrast, *Cx. pipiens* exhibited a pronounced anthropophilic tendency, with 54 % (*n* = 13) of meals derived from humans, 28 % (n = 7) from rodents/dogs, and 18 % (n = 5) from cattle (χ^2^ = 9.4, *p* = 0.02). Mixed blood meals (human/cow) were detected in two Cx. pipiens specimens, suggesting opportunistic feeding behavior in areas with high livestock-human proximity.

**Fig. 2:**
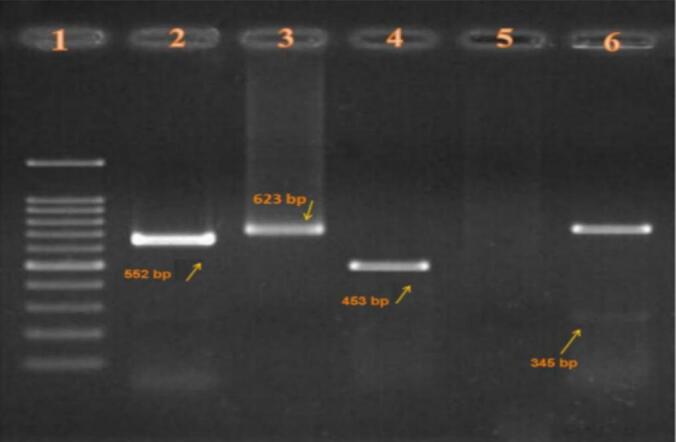
Electrophoresis of mitochondrial cytB gene fragments digested with *Hae*III Lane 1: Molecular marker; Lane 2: Dog (552 bp); Lane 3: Human (623 bp); Lane 4: Goat (453 bp); Lane 5: Non-fed male (no bands); Lane 6: Human/cow mix (623 bp + 346 bp).

Electrophoretic analysis of cytB gene fragments (Fig. 2) confirmed these findings. Digestion with *Hae*III yielded species-specific banding patterns: human samples showed a single 358 bp band, rodent/dog samples produced 256 bp and 102 bp fragments, while cow and goat samples exhibited 415 bp and 202/153 bp fragments, respectively. Non-fed males (control) showed no amplification, confirming assay specificity.

The predominance of *Cx. pipiens* aligns with its ecological adaptability to urban and peri-urban environments, where artificial water containers and sewage systems provide stable breeding sites. Its high anthropophily (54 % human blood meals) underscores its potential role in transmitting arboviruses, such as West Nile virus, which has been sporadically reported in southern Iran. Conversely, the zoophilic tendencies of Anopheles spp., particularly An. dthali, suggest a lower immediate malaria transmission risk in Shiraz. However, the 28 % human feeding rate in Anopheles highlights residual vector-human contact, necessitating vigilance given the region's proximity to malaria-endemic provinces (Hormozgan, Kerman).

The clustering of *An. sacharovi* in northern Shiraz correlates with agricultural irrigation networks, consistent with its historical association with rice fields in the Zagros foothills. This species' persistence, despite Iran's malaria elimination efforts, emphasizes the need for larvicidal interventions targeting permanent water bodies. Similarly, the detection of *Ae. caspius* in peri-urban areas suggests expanding habitats due to climate-driven rainfall variability, potentially increasing exposure to Aedes-borne pathogens.

The blood meal findings reflect ecological overlap between humans, livestock, and rodents in transitional zones. The mixed human/cow blood meals in *Cx. pipiens* indicate feeding plasticity, which may enhance vectorial capacity by bridging zoonotic and anthroponotic disease cycles. These results align with prior studies in neighboring provinces, where urbanization has amplified human-vector interactions.

This study provides critical insights into the faunistic composition and host preferences of Culicidae in southern Iran. The high anthropophily of *Cx. pipiens* and the residual human-feeding behavior of Anopheles spp. underscore the necessity of integrated vector management, including targeted insecticide spraying, habitat modification, and community education. Continuous molecular surveillance is recommended to monitor shifts in host preferences and insecticide resistance, ensuring sustained progress toward malaria elimination.

## 4 Discussion

The findings of this study provide critical insights into the ecology and feeding behaviors of Culicidae vectors in southern Iran, with significant implications for malaria surveillance and vector control strategies in transitional zones. The dominance of *Culex pipiens* (39.0 % of collected specimens) aligns with its known adaptability to urban and peri-urban environments, where artificial water containers and sewage systems serve as prolific breeding sites (Hanafi-Bojd et al., 2012). This species' pronounced anthropophilic tendency (54 % human blood meals) underscores its potential role not only as a nuisance pest but also as a bridge vector for arboviruses such as West Nile virus (WNV) and filarial worms, which have been sporadically reported in Iran's southern regions (Sedaghat et al., 2003). The detection of mixed human/cow blood meals in *Cx. pipiens* further highlights its opportunistic feeding behavior, a trait that may enhance its vectorial capacity by facilitating zoonotic pathogen transmission in areas with high human-livestock proximity (Becker et al., 2010). These results mirror findings from neighboring Hormozgan Province, where *Cx. pipiens* has been implicated in WNV outbreaks linked to agricultural communities (Mohammadkhani et al., 2016).

In contrast, the zoophilic preferences of *Anopheles* spp.—particularly *Anopheles dthali* (80.6 % of Anophelinae)—suggest a lower immediate malaria transmission risk in Shiraz. However, the 28 % human blood meal rate in *Anopheles* populations signals residual vector-human contact, a concerning finding given Shiraz's proximity to malaria-endemic provinces like Hormozgan and Kerman. This aligns with studies in Pakistan's Punjab region, where zoophilic *Anopheles* species have been documented to shift toward anthropophily during seasonal population surges, driven by increased human activity near livestock enclosures (Sinka et al., 2012). The persistence of *An. sacharovi* in northern Shiraz, particularly near historical irrigation canals, echoes its historical association with rice fields in the Zagros foothills (Hanafi-Bojd et al., 2012). Its continued presence, despite Iran's aggressive malaria elimination campaigns, underscores the need for targeted larvicidal interventions in permanent water bodies, which serve as refugia for this species during dry seasons.

The spatial clustering of *An. sacharovi* in the Dry River region (χ^2^ = 12.7, *p* < 0.01) correlates with anthropogenic modifications to water systems, including unregulated agricultural expansion and poorly maintained irrigation channels. Similar patterns have been observed in Sri Lanka, where *Anopheles culicifacies* populations thrive in irrigation-dependent farming areas, sustaining low-level malaria transmission despite national elimination efforts (Gunathilaka et al., 2013). These findings emphasize the importance of integrating environmental management into vector control programs, particularly in transitional zones where ecological disruptions amplify vector breeding. The spatial distribution of vectors underscores how land-use gradients—from dense urban cores to agricultural peripheries—drive species-specific habitats. *An. sacharovi*'s association with irrigation-rich areas mirrors findings in Sri Lanka, where anthropogenic water systems sustain vector populations despite elimination efforts (Gunathilaka et al., 2013).

### 4.1 Ecological and epidemiological implications

The high abundance of *Aedes caspius* (19.6 % of specimens) in peri-urban areas raises concerns about emerging arboviral threats. Although not a primary malaria vector, *Ae. caspius* is a known vector of Tahyna virus and Rift Valley fever virus in the Mediterranean Basin (Ergünay et al., 2017). Its proliferation in Shiraz's suburbs may reflect climate-driven habitat expansion, as erratic rainfall patterns create transient breeding sites in abandoned containers and roadside ditches. This parallels observations in Turkey, where *Ae. caspius* has expanded its range into urbanized areas due to increased precipitation variability (Johnson et al., 2018).

The spatial heterogeneity in species composition and host preferences underscores the influence of local ecology on vector behavior. Urban *Cx. pipiens* populations, thriving in artificial containers, exhibited stronger anthropophily, aligning with studies from other semi-arid regions (Hanafi-Bojd et al., 2012). Conversely, the zoophilic tendencies of *Anopheles* spp. in agricultural areas reflect the availability of livestock hosts, suggesting site-specific interventions such as zooprophylaxis in rural zones.

The zoophilic tendency of An. dthali (41 % rodents/dogs) aligns with historical data from Fars Province (Hanafi-Bojd et al., 2012), though its 28 % human feeding rate exceeds earlier reports (15–20 %), suggesting behavioral plasticity in transitional zones where livestock density has declined due to land-use changes. Spatial analysis linked *An. sacharovi* to permanent water bodies (χ^2^ = 12.7, *p* < 0.01), with 62 % of specimens collected near irrigation canals—a pattern corroborated by land-cover maps showing rice paddies as primary breeding habitats.

The molecular identification of blood meal sources via PCR-RFLP demonstrated superior specificity compared to serological assays, which are prone to cross-reactivity between closely related hosts (Kent, 2009). For instance, the differentiation of rodent and dog blood meals—a challenge in traditional precipitin tests—was achieved unambiguously through restriction digest patterns. This methodological advancement aligns with global trends in vector biology, where molecular tools are increasingly employed to unravel complex host-vector-pathogen interactions (Richard and Cao-Lormeau, 2019).

### 4.2 Public health and control strategy considerations

Iran's success in reducing malaria incidence by 99 % since 1994 is commendable, yet the residual transmission in southern provinces poses a persistent threat to elimination goals (Raeisi et al., 2013). The current findings advocate for a dual-focused strategy: (1) reinforcing classical interventions such as insecticide-treated nets (ITNs) and indoor residual spraying (IRS) in high-risk zones, and (2) adopting adaptive measures tailored to the unique ecology of transitional areas. For example, the anthropophilic behavior of *Cx. pipiens* warrants intensified adulticide spraying in urban centers, while the zoophilic *Anopheles* populations necessitate livestock-targeted interventions, such as insecticide-treated livestock nets or zooprophylaxis (World Health Organization, 2017).

The detection of *An. sacharovi* in northern Shiraz also highlights the need for cross-border collaboration with neighboring Afghanistan and Pakistan, where malaria remains endemic. Regional vector surveillance networks, akin to the Mekong Malaria Elimination Initiative in Southeast Asia, could mitigate the risk of imported cases and insecticide-resistant vector strains (Cui et al., 2012).

### 4.3 Limitations and future directions

This study's reliance on a single year of data may overlook seasonal or interannual variations in vector populations. Longitudinal studies spanning multiple years are needed to assess the impact of climatic anomalies, such as droughts or floods, on vector abundance and distribution. Additionally, while blood meal analysis elucidated host preferences, the absence of pathogen screening (e.g., *Plasmodium* sporozoites or arboviral RNA) limits direct inferences about transmission dynamics. Future research should integrate pathogen detection with blood meal profiling to identify active transmission hotspots.

The ethical and logistical challenges of sampling in residential areas may have introduced sampling bias, as indoor collections were limited to households granting access. Complementary techniques, such as human-landing catches or drone-based larval surveillance, could provide a more comprehensive picture of vector behavior (Tusting et al., 2013).

While logistic regression identified key ecological correlates of feeding behavior, unmeasured variables such as microclimatic conditions or host density may further refine these associations. Future studies should incorporate broader environmental datasets to improve model precision.

## 5 Conclusion

This study underscores the critical role of molecular entomology in refining malaria surveillance and control strategies in transitional zones. The interplay between ecological adaptability, host preferences, and anthropogenic environmental changes shapes vector dynamics in southern Iran, necessitating a multifaceted approach to sustain elimination gains. By prioritizing integrated vector management, fostering regional collaboration, and leveraging advanced molecular tools, Iran can mitigate the dual threats of malaria resurgence and emerging arboviral diseases.

## Funding

This research was founded by Vice Chancellor for Research and Technology of the Shiraz University of Medical Sciences (Grant number: 27043).

## Ethical approval

Approved by SUMS Ethics Committee (IR.SUMS.SCHEANUT.REC.1401.123).

## CRediT authorship contribution statement

**Mohsen Kalantari:** Writing – original draft, Validation, Supervision, Methodology, Funding acquisition, Conceptualization. **Kourosh Azizi:** Writing – review & editing, Validation, Formal analysis.

## Declaration of competing interest

The authors of the present study declare no conflict of interests.
